# Draft Genome Sequences of Two Multidrug-Resistant *Acinetobacter baumannii* Strains of Sequence Type ST92 and ST96

**DOI:** 10.1128/genomeA.00296-13

**Published:** 2013-05-30

**Authors:** Alex Y. M. Ho, Kin-Hung Chow, Pierra Y. T. Law, Herman Tse, Pak-Leung Ho

**Affiliations:** Department of Microbiology, University of Hong Kong, Hong Kong SAR, China

## Abstract

The global epidemiology of multidrug-resistant *Acinetobacter baumannii* is dominated by a limited number of clones. Here, we announce the draft genome sequences of two multidrug-resistant *A. baumannii* strains, 1H8 and 4A3, representing the major epidemic clones, sequence type 92 (ST92) and ST96, respectively.

## GENOME ANNOUNCEMENT

*Acinetobacter baumannii* is a major nosocomial pathogen that displays a remarkable ability to acquire mechanisms that confer resistance to virtually all antibiotics (1, 2). Analysis of the population structure of *A. baumannii* has shown that the global emergence of multidrug resistance in this organism is predominated by a number of widely distributed clones (2). In Hong Kong hospitals, the increase in multidrug-resistant *A. baumannii* is mainly attributed to the expansion of two clones, sequence type 92 (ST92) and ST96 (3, 4). ST92 is a pandemic clone with distribution in >30 countries (2), while ST96 is a recently identified clone that has so far been geographically restricted to certain parts of China (2, 4).

In this report, we present the draft genomes of two *A. baumannii* strains, 1H8 (ST92) and 4A3 (ST96), originating from clinical samples of two hospitalized patients in Hong Kong, China (4). The bacterial genomes were sequenced on a GS FLX system (Roche Diagnostics, Basel, Switzerland). A total of 263,241 reads from strain 4A3 (ST96) and 247,690 reads from strain 1H8 (ST92) were generated at approximately 23-fold genome coverage for both strains*. De novo* genome assembly was performed using the Newbler assembler 2.7 (Roche Diagnostics). The contigs were then oriented and ordered into scaffolds using OSLay (5). Gene identification and automatic functional annotation were performed using the RAST (Rapid Annotations using Subsystem Technology) server and Blast2GO (6, 7). The antibiotic resistomes in the genomes were identified using the Antibiotic Resistance Genes Database (8).

The genome assembly of strain 4A3 (ST96) consists of 136 contigs, with a G+C content of 38.9%, a total length of 4,104,065 bp, and 3,916 protein-coding sequences. The genome assembly of strain 1H8 (ST92) consists of 69 contigs, with a G+C content of 39.0%, a total length of 3,993,780 bp, and 3,774 protein-coding sequences. In the genomes of strain 4A3 (ST96) and strain 1H8 (ST92), there were 41 and 49 antimicrobial resistance genes, respectively. These include efflux pumps (ABC, major facilitator superfamily [MFS], small multidrug resistance [SMR], and resistance-nodulation-division [RND] families) potentially carrying resistance to a wide range of compounds and genes associated with specific resistance to β-lactams (class A, C, and D β-lactamases), chloramphenicol (*cat*, *cml*), aminoglycosides (phosphotransferases, adenyltransferases, nucleotidyltransferases, and dimethyladenosine transferases), macrolides-lincosamides-streptogramins (*macB*, *mph2*, *msrA*, *vat*, *vgaA*), tetracyclines (*tetB*), trimethoprim (*dhfr*), and sulphonamides (*sul*). Overall, the availability of the present genome sequences facilitates further comparative genomic and bioinformatics analysis in *A. baumannii* populations.

### Nucleotide sequence accession numbers.

This Whole-Genome Shotgun project has been deposited at NCBI GenBank under the accession no. AOLU00000000 (4A3) and ANNC00000000 (1H8). The genome sequences described in this paper are the first versions, AOLU01000000 (4A3) and ANNC01000000 (1H8).
